# Safety and efficacy of Pucotenlimab (HX008) - a humanized immunoglobulin G4 monoclonal antibody in patients with locally advanced or metastatic melanoma: a single-arm, multicenter, phase II study

**DOI:** 10.1186/s12885-022-10473-y

**Published:** 2023-02-06

**Authors:** Chuanliang Cui, Yu Chen, Zhiguo Luo, Zhengyun Zou, Yu Jiang, Hongming Pan, Qingxia Fan, Jianfu Zhao, Qing Xu, Renbing Jiang, Xuan Wang, Taiyang Ma, Zhen Guo, Lu Si, Zhihong Chi, Xinan Sheng, Yiwei Dou, Qian Tan, Di Wu, Jun Guo

**Affiliations:** 1 Key Laboratory of Carcinogenesis and Translational Research (Ministry of Education/Beijing), Department of Renal Cancer and Melanoma, Peking University Cancer Hospital & Institute, Beijing, China; 2 Fujian Cancer Hospital, Fuzhou, China; 3 Fudan University Shanghai Cancer Center, Shanghai, China; 4 Drum Tower Hospital, Affiliated to Medical School of Nanjing University, Nanjing, China; 5 West China Hospital, Sichuan University, Chengdu, China; 6 Zhejiang University School of Medicine Sir Run Run Shaw Hospital, Hangzhou, China; 7 The First Affiliated Hospital of Zhengzhou University, Zhengzhou, China; 8 The First Affiliated Hospital of Jinan University, Guangzhou, China; 9 Shanghai Tenth People’s Hospital, Shanghai, China; 10 The Affiliated Cancer Hospital of Xinjiang Medical University, Ürümqi, China; 11 Taizhou Hanzhong Biomedical Co., Ltd. (A Member of Lepu Biopharma Co., Ltd.), Taizhou, China; 12 The First Hospital of Jilin University, Changchun, China

**Keywords:** PD-1, Monoclonal antibody, Pucotenlimab, HX008, Melanoma

## Abstract

**Background:**

Pucotenlimab is a novel recombinant humanized anti-PD-1 (Programmed death-1) monoclonal antibody, which belongs to the human IgG4/kappa subtype, and can selectively block the binding of PD-1 with its ligands PD-L1 and PD-L2.

**Methods:**

In this phase 2 trial, patients with locally advanced or metastatic melanoma who had failed conventional treatment (chemotherapy, targeted therapy, interferon, IL-2, et al.) were recruited. The patients were administrated with Pucotenlimab of 3 mg/kg every 3 weeks until disease progression, intolerable toxicity, or treatment discontinuation for any other reasons. The primary endpoint was the overall response rate (ORR). The secondary endpoints were disease control rate (DCR), duration of response (DOR), progression-free survival (PFS), overall survival (OS), and toxicity.

**Results:**

One-hundred and nineteen patients were enrolled and followed up for 19.32 (ranging from 15.901 to 24.608) months by the cutoff date of July 30^th^, 2021. The ORR was 20.17% (24/119, 95% CI, 13.370%-28.506%) based on both independent review committee (IRC) and the investigator’s assessment per RECIST v1.1. The median PFS were 2.89 (95% CI, 2.037–4.074) months and 2.46 (95% CI, 2.004–4.008) months based on IRC and investigator’s assessment, respectively, per RECIST v1.1. The median OS was 16.59 (95% CI, 13.963–26.973) months. Treatment-related adverse events (TRAEs) occurred in 77.3% (92/119) of the patients. The incidence of Grade ≥ 3 TRAEs was 15.1% (18/119). In addition, none of the patients died because of TRAEs. As for biomarker analysis, Eotaxin (CCL11) and MCP-1 (CCL2) were related to treatment response, while TNF-α and VEGF were related to treatment failure.

**Conclusions:**

Pucotenlimab as a ≥ 2^nd^ line therapy showed promising efficacy and tolerable toxicity for patients with locally advanced or metastatic melanoma.

**Trial registration:**

Clinicaltrials.gov Identifier: NCT04749485 (registered retrospectively on 11/02/2021).

## Background

The incidence of melanoma has rapidly increased in the past decades [1, 2]. According to global cancer statistics in 2018 [2], more than 280,000 new cases of cutaneous melanoma and more than 60,000 related deaths were estimated worldwide, accounting for 1.6% and 0.6% of the total number of new cancer cases and cancer-related deaths, respectively. Among the white population, 90% of melanoma originated from the skin [3]. While for Asians, 58% of melanoma patients have an acral or mucosal origin [4]. In China, the incidence of melanoma is low, with approximately 20,000 cases reported annually. But unfortunately, the population suffered is increasing rapidly with an annual rate of 3%-5% [5].

Currently, early-stage melanoma is mainly treated with surgery with a good prognosis. However, before anti-Cytotoxic T-lymphocyte antigen 4 (CTLA-4) or PD-1 antibodies were available, the treatment options for advanced melanoma were limited and usually with frustrating outcomes. Among those, dacarbazine was the optimal one. The median OS was less than 1 year, and ORR was about 5% [6]. While, in the era of immunotherapy, PD-1 blockade significantly improved the median OS, PFS, and ORR of various malignant tumors [7]. For the indication of unresectable or metastatic malignant melanoma, Nivolumab and Pembrolizumab as single agents were approved by the US Food and Drug Administration in 2014 [8], and Nivolumab plus Ipilimumab as a combined therapy was approved in 2015 [9]. In 2018, the Chinese National Medical Products Administration approved Pembrolizumab and Toripalimab for treating previously treated unresectable or metastatic melanoma [10]. Due to the rarity of acral and mucosal melanoma, especially in the Caucasian population, and the different biological features, the efficacies of the approved immune checkpoint inhibitors reported in acral and mucosal melanoma were underexplored and inconsistent compared with those reported in cutaneous melanoma. Based on a series of clinical trials with small sample sizes, the ORRs were 11.4 ~ 34% and 3.9 ~ 35% for acral melanoma and mucosal melanoma, respectively, and the corresponding median PFS were 2.1 ~ 6.6 months and 1.4 ~ 9 months [11]. Pucotenlimab is a novel, fully-humanized monoclonal antibody which selectively blocks the interaction between PD-1 and its ligands [12]. Here, we report the safety and efficacy of Pucotenlimab in the Chinese population with locally advanced or metastatic melanoma who had failed the standard treatment (chemotherapy, targeted therapy, interferon, IL-2, et al.).

## Methods

### Study design and participants

This clinical trial is a multi-center, open-label, single-arm phase II study. Eligible patients were aged 18 to 75 with a histologic diagnosis of unresectable locally advanced or metastatic melanoma and must have failed at least 1 prior routine regimen for the advanced disease. The number of patients with primary mucosal melanoma should be less than 22%. The Eastern Cooperative Oncology Group (ECOG) performance status of the patients must be scored 0 or 1, and with a life expectancy ≥ 3 months. There must be at least 1 measurable extracranial lesion based on RECIST v1.1 [13] without prior radiation. The central nervous system metastases must be asymptomatic and be stable for at least 3 months. The patients must have sufficient organ and bone marrow function based on the following laboratory examination standards: neutrophils ≥ 1.5 × 10^9/L; white blood cells ≥ 3.0 × 10^9/L; platelets ≥ 100 × 10^9/L; hemoglobin ≥ 90 g/L; serum creatinine ≤ 1 × ULN; aspartic transaminase (AST) and alanine transaminase (ALT) ≤ 2.5 × ULN without, and ≤ 5 × ULN with hepatic metastasis; total bilirubin ≤ 1.5 × ULN; INR ≤ 2 × ULN, aPTT ≤ 1.5 × ULN (except for those undergoing anticoagulant therapy). Patients with reproductive potential should be willing to take adequate contraceptive measures from enrollment to 3 months after the last administration of the trial drug.

The major exclusion criteria included: Melanoma of ocular origin; Prior treatments contained anti-PD-1/PD-L1/CTLA-4 antibody; Patients with active or history of autoimmune diseases that may recur, or with high risk; Expecting to receive major surgery during the study period; Needing to receive systemic corticosteroids (dose equivalent to > 10 mg prednisone/day) or other immunosuppressive drugs within 14 days before enrollment or during the study period; history of human immunodeficiency virus infection, acquired or congenital immunodeficiency disease, organ transplantation or stem cell transplantation; having active chronic HBV or HCV infection, except those with HBV DNA viral load ≤ 500 IU/mL or < 10^3 copies/mL, or HCV RNA negative after adequate treatment; Known to be allergic to macromolecular protein agents or monoclonal antibody; Have participated in other clinical trial within 4 weeks prior to the first dose of the study drug.

Eligible patients received Pucotenlimab 3 mg/kg by intravenous infusion over 60 min on day 1, every 3 weeks until disease progression, intolerable toxicity, or treatment discontinuation for any other reasons.

### Endpoints and assessments

Tumor response was assessed every 9 weeks for 3 times and every 12 weeks thereafter. The primary endpoint was ORR assessed by IRC based on RECIST v1.1. The secondary endpoints were ORR assessed by investigators based on iRECIST [14]; DCR, DOR, and PFS assessed by IRC and investigator based on RECIST v1.1 and iRECIST; OS, and the safety. The safety evaluations were performed among patients who received at least one dose of the study treatment, and the severity of adverse events was graded according to CTCAE v5.0. The potential anti-drug-antibody (ADA) was continuously monitored in the blood samples collected prior to each dosing of Pucotenlimab. Survival was followed up every 12 weeks after discontinuation. In order to identify potential biomarkers associated with efficacy, cytokines in blood samples from 8 patients at C2D1 (Day 1 of treatment cycle 2), C3D1, C5D1, C7D1, C9D1, C11D1, C13D1, C15D1, C17D1 were measured via Bead-Based Multiplex Assays using the Luminex technology. These cytokines included EGF, FGF-2, Eotaxin, TGF-α, GRO, MDC, IL-5, IL-8, IP-10, MCP-1, MIP-1α, MIP-1β, TNF-α, and VEGF. The relationships between the levels of these cytokines and the corresponding response status (i.e., complete response/CR, partial response/PR, stable disease/SD, progressed disease/PD) were analyzed as exploratory endpoints.

### Subgroup analysis

In order to explore potential prognostic factors for efficacy, the ORRs of the patients grouped by various baseline characteristics was compared retrospectively, which included age, gender, ECOG PS, melanoma subtypes, distant metastasis, ADA, liver metastasis, BRAF mutation, NRAS mutation, and baseline LDH.

### Statistical assessments

Assuming a targeted ORR of 20%, a total of 109 patients could provide 90% confidence in the unilateral 5% alpha level to prove that Pucotenlimab is superior to the historical control of 10% [6]. Considering a dropout rate of about 10%, 122 subjects would be enrolled.

The full analysis set (FAS) included all the patients who were administrated with at least one dose of Pucotenlimab. Efficacy and Safety analysis will be conducted based on FAS. ORR and DCR with 95% CI were calculated using the Clopper–Pearson exact method based on binomial distribution. Patients without tumor assessment data were considered as non-respond or rather, PD. Kaplan–Meier method was used to estimate median DOR and PFS, and their 95% CIs were estimated by Brookmeyer-Crowley method. Safety was analyzed using descriptive statistics. Data analyses were conducted using SAS statistical software version 9.4 and MedCalc Version 20.2.

## Results

### Patient population

In this study, a total of 164 patients were selected and 119 patients were enrolled at 11 centers in China, of whom 42.9% were males and 57.1% were females. Their median age was 59 (23–74) years. ECOG PS in 42.9% of the patients were scored 0, and the rest were scored 1. The proportion of primary cutaneous subtype was 18.5%, acral subtype was 52.1%, mucosal subtype was 19.3%, and those with unknown primary site was 10.1%. Melanoma in 22.7% of the patients was stage III, and 72.9% were stage IV. Seven point six percent of the patients had NRAS gene mutation, and 10.9% had BRAF gene mutation. Patients received 1, 2, or ≥ 3 lines of previous treatments were 63.9%, 28.6%, and 7.6%, respectively. Baseline characteristics are listed in Table 1. From November 08, 2017, to June 30^th^, 2021, the patients were followed up for 19.32 (range, 0.99–31.34) months.

**Table 1:** Summary of patient characteristics

Characteristic	Value	N (%) *N* = 119
Gender	Male	51 (42.9)
	Female	68 (57.1)
Age, years	Mean (SD)	57.5 (9.41)
	Median	59.0
	Range	23 to 74
ECOG performance status	0	51 (42.9)
	1	68 (57.1)
Melanoma subtypes	Non-acral cutaneous	22 (18.5)
	acral	62 (52.1)
	mucosal	23 (19.3)
	unknown primary	12 (10.1)
Metastatic stage	M0	27 (22.7)
	M1	40 (33.6)
	M1a	10 (8.4)
	M1b	26 (21.9)
	M1c	12 (10.1)
	unknown	16 (13.4)
Clinical stage	II	1 (0.8)
	III	27 (22.7)
	IV	86 (72.3)
	Unknown	5 (4.2)
Type of prior therapy	Surgery	101 (84.9)
	Radiotherapy	11 (9.2)
	Chemotherapy	83 (69.7)
	Target therapy	18 (15.1)
	Immunotherapy (IFN-α 2b, IL-2, oncolytic viruses, CIK, NKT, et al.)	52 (43.7)
	Biotherapy	11 (9.2)
	Others	36 (30.3)
Prior lines of treatment for advanced disease	1	76 (63.9)
	2	34 (28.6)
	≥ 3	9 (7.6)
liver metastasis	YES	15 (12.6)
	NO	104 (87.4)
NRAS gene mutation	Positive	9 (7.6)
	Negative	1 (0.8)
	Unknown	109 (91.6)
BRAF gene mutation	Positive	13 (10.9)
	Negative	47 (39.5)
	Unknown	59 (49.6)
LDH level	Normal	72 (60.5)
	Higher than upper limit of normal (ULN)	47 (39.5)

### Efficacy

The IRC assessed ORRs were 20.17% (95% CI, 13.370%-28.506%) and 21.85% (95% CI, 14.796%-30.352%) according to RECIST v1.1 and iRECIST, and 20.17% (95% CI, 13.370%-28.506%) and 22.69% (95% CI, 15.516%-31.268%) based on the investigator’s assessment (Table 2). As the lower limit of the 95% CI of actual ORR exceeded the threshold of 10%, the primary endpoint of this study was reached.

**Table 2:** Clinical efficacy evaluated by independent review committee (IRC) and investigator per RECIST v1.1 or iRECIST

	**IRC**		**Investigator**	
N	119		119	
	RECIST	iRECIST	RECIST	iRECIST
CR/iCR, n (%)	1 (0.84)	3 (2.52)	2 (1.86)	2 (1.68)
PR/iPR, n (%)	23 (19.33)	23 (19.33)	22 (18.49)	25 (21.01)
SD/iSD, n (%)	28 (23.53)	31 (26.05)	27 (22.69)	28 (23.53)
PD/iuPD/icPD, n (%)	57 (47.90)	52 (43.7)	58 (48.74)	54 (45.4)
NE, n (%)	10 (8.4)	10 (8.4)	10 (8.4)	10 (8.4)
ORR, n (%)	24 (20.17)	26 (21.85)	24 (20.17)	27 (22.69)
95% CI	13.370–28.506	14.796–30.352	13.370–28.506	15.516–31.268
DCR, n (%)	52 (43.70)	57 (47.90)	51 (42.86)	55 (46.22)
95% CI	34.625–53.091	38.658–57.248	33.827–52.252	37.037–55.592
Median PFS, months (95% CI)	2.89 (2.037–4.074)	4.01 (2.201–4.665)	2.46 (2.004–4.008)	3.98 (2.333–6.012)
cutaneous	4.205 (2.037–13.142)	_	_	_
acral	3.285 (2.004–4.107)	_	_	_
mucosal	1.971 (1.873–2.661)	_	_	_
Median DOR, months (95% CI)	NR (9.791, NR)	NR (17.577, NR)	NR (15.869, NR)	NR (15.869, NR)
12-month DOR rate, % (95% CI)	68.46 (42.36–84.60)	80.36 (55.64–92.17)	81.99 (58.83–92.83)	83.27 (61.29–93.38)
Median OS, months (95% CI)	16.59 (13.963–26.973)			
cutaneous	NR			
acral	20.107 (14.193–27.039)			
mucosal	9.363 (5.092–22.505)			

*NE* refers to the patients who discontinued before the first efficacy evaluation. NR means not reached

As for subgroup analysis, the IRC assessed ORRs for four subtypes of melanoma, ie, cutaneous, acral, mucosal, and unknown primary site were 36.36% (95% CI, 17.198%-59.342%), 16.13% (95% CI, 8.015%-27.668%), 8.70% (95% CI, 1.071%-28.038%), and 33.33% (95% CI, 9.925%-65.112%), respectively. Four Patients with positive ADA showed no response to Pucotenlimab treatment. While 112 patients of ADA-negative showed an ORR of 21.43% (95% CI, 14.24%-30.19%). Five out of 13 patients with BRAF mutations and 1 out of 9 with NRAS mutation responded to Pucotenlimab treatment. On the other hand, 8 out of 47 and 0 out of 1 patient without BRAF or NRAS mutation demonstrated response to Pucotenlimab. The comprehensive information was shown in Table 3.

**Table 3:** ORRs of different subgroups assessed by IRC per RECIST v1.1

Subgroup		Response/Patients	ORR, % (95%CI)
Overall		24/119	20.17 (13.37–28.51)
Age	< 65	15/92	16.30 (9.42–25.46)
	≥ 65	9/27	33.33 (16.52–53.96)
Gender	Male	9/51	17.65 (8.40–30.87)
	Female	15/68	22.06 (12.90–33.76)
ECOG PS	0	11/51	21.57 (11.29–35.32)
	1	13/68	19.12 (10.59–30.47)
Melanoma subtypes	Non-acral cutaneous	8/22	36.36 (17.20–59.34)
	Acral	10/62	16.13 (8.02–27.67)
	Mucosal	2/23	8.70 (1.07–28.04)
	Unknown	4/12	33.33 (9.92–65.11)
Distant metastasis	No	3/27	11.11 (2.35–29.16)
	Yes	17/76	22.37 (13.60–33.38)
ADA	Positive	0/4	0
	Negative	24/112	21.43 (14.24–30.19)
Liver metastasis	Yes	1/15	6.67 (0,17–31.95)
	No	23/104	22.12 (14.57–31.31)
BRAF mutation	Yes	5/13	38.46 (13.86–68.42)
	No	8/47	17.02 (7.65–30.81)
NRAS mutation	Yes	1/9	11.11 (0.28–48.25)
	No	0/1	0
Baseline LDH	Normal	18/72	25.00 (14.75–35.25)
	Higher than upper limit of normal (ULN)	6/47	12.77 (2.86–22.67)

**Fig. 1:**
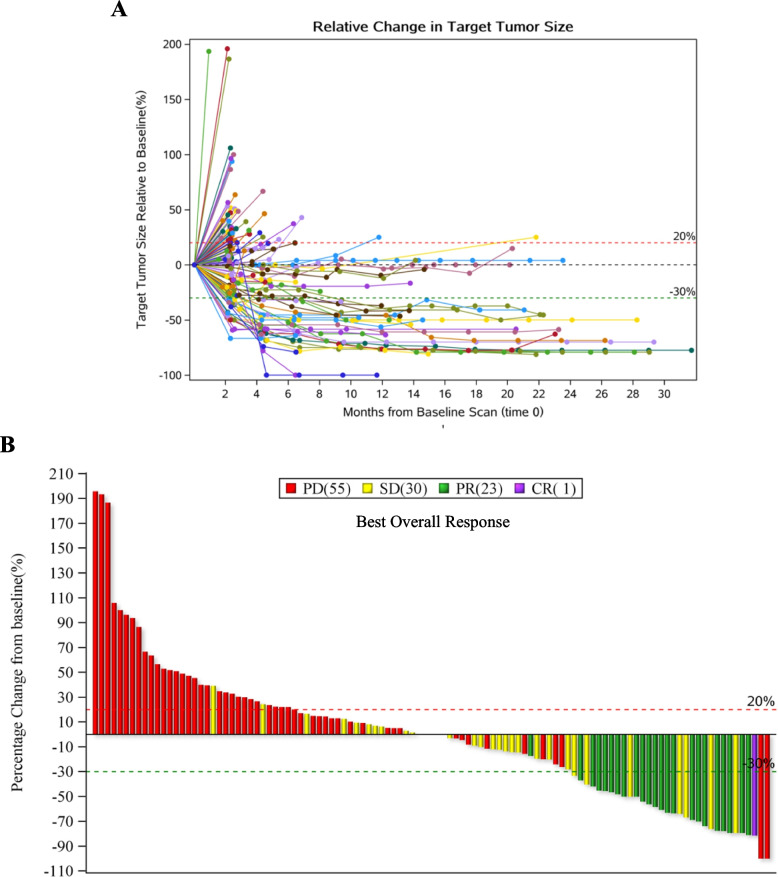
The Changes in target lesions size. **A** Changes of target lesion size at different evaluation time points compared to the baseline for the patients whose efficacy data were available (*N* = 109). **B** Best percentage change in target lesion size from baseline based on IRC assessment per RECIST v1.1 for the patients whose efficacy data were available (*N* = 109)

**Fig. 2:**
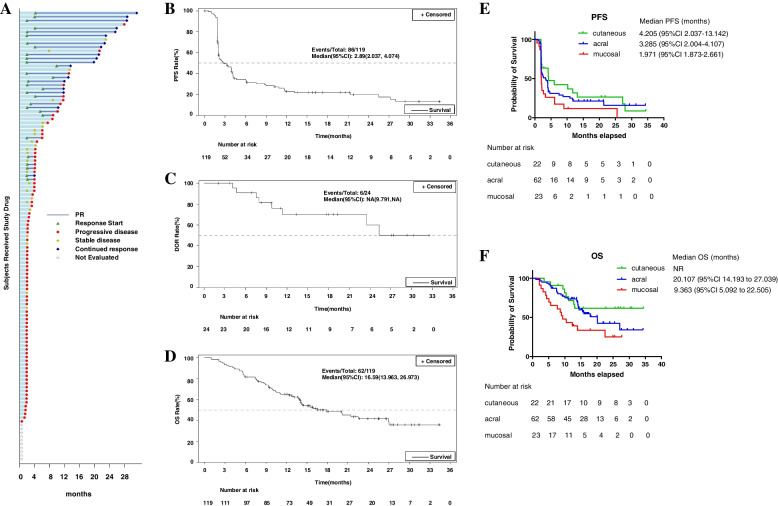
DOR, PFS, and OS for the patients involved. **A** Duration of response for each individual patients based on IRC assessment per RECIST v1.1 (*N* = 119). **B** Kaplan–Meier estimates of PFS in the full-analysis set based on IRC assessment per RECIST v1.1. **C** Kaplan–Meier estimates of DOR for the patients achieved PR/CR based on IRC assessment per RECIST v1.1 (*N* = 24). **D** Kaplan–Meier estimates of OS in the full-analysis set. **E** Kaplan–Meier estimates of PFS for different primary subtypes based on IRC assessment per RECIST v1.1. **F** Kaplan–Meier estimates of OS for different primary subtypes

The IRC assessed PFS, DOR, and 12-month DOR rate per RECIST v1.1 were 2.89 (95% CI, 2.037–4.074) months, not reached, and 68.46% (95% CI, 42.36%-84.60%), respectively. The OS was 16.59 (95% CI, 13.963,26.973) months. Especially, the IRC assessed median PFS 4.205 (95% CI, 2.037–13.142), 3.285 (95% CI, 2.004–4.107) and 1.971 (95% CI, 1.873–2.661) months for cutaneous, acral, and mucosal subtypes, respectively; and their corresponding median OS were NR, 17.91 (95% CI, 13.832 to 20.107), and 9.363 (95% CI, 5.092 to 10.480) months, respectively. The detailed efficacy parameters were listed in Table 2. The Changes in target lesions size were showed in Fig. 1A-B. The Kaplan–Meier estimates of PFS, DOR, and OS were demonstrated in Fig. 2A-F. During the survival follow-up period after Pucotenlimab failure, 55 patients applied other treatments, including chemotherapies, target therapies, anti-angiogenesis therapies, PD-1 + chemo or targeted therapies, or oncolytic virus. Merely 2 patients achieved SD, and none achieved CR or PR from the follow-up treatments.

### Safety

For the 119 patients who received at least one dose of Pucotenlimab, 98.3% reported TEAEs, 77.3% reported TRAEs, and 15.1% reported TRAEs of Grade ≥ 3. TRAEs of occurrence ≥ 10% were thyroid function test abnormal (26.1%), skin depigmentation (21.0%), rash (16.8%), aspartate aminotransferase increased (15.1%), hyperlipidaemia (13.4%), blood bilirubin increased (12.6%), alanine aminotransferase increased (12.6%), and hypothyroidism (12.6%) as shown in Table 4. SAEs, treatment-related SAEs, and irAEs were reported in 21.0%, 9.2%, and 47.9% of the patients. irAEs reported in ≥ 10% of patients were skin depigmentation (15.1%), aspartate aminotransferase increased (10.1%), rash (10.1%) and hypothyroidism (10.1%). TRAEs that led to treatment interruption or treatment discontinuation were14.3%, 6.7%, respectively, and none of the patients died because of TRAEs.

**Table 4:** Safety profile of the patients who were given at least one dose of Pucotenlimab (*N* = 119)

All patients (*N* = 119)	Incidence (%)	
TEAEs	98.3	
TRAEs	77.3	
TRAEs reported in ≥ 10% of patients	Any Grade	Grade ≥ 3
Thyroid function test abnormal	26.1	0
Skin depigmentation	21.0	0
Rash	16.8	0.8
Aspartate aminotransferase increased	15.1	1.7
Hyperlipidaemia	13.4	2.5
Blood bilirubin increased	12.6	1.7
Alanine aminotransferase increased	12.6	0
hypothyroidism	12.6	0
SAEs	21.0	
Treatment-related SAEs	9.2	
TEAEs of Grade ≥ 3	34.5	
TRAEs of Grade ≥ 3	15.1	
TEAEs leading to treatment interruption	21.8	
TRAEs leading to treatment interruption	14.3	
TEAEs leading to treatment discontinuation	18.5	
TRAEs leading to treatment discontinuation	6.7	
irAEs	47.9	
irAEs reported in ≥ 1% of patients	Any Grade (%)	
Skin depigmentation	15.1%	
Aspartate aminotransferase increased	10.1%	
Rash	10.1%	
Hypothyroidism	10.1%	
irSAEs	9.2%	

### Biomarker analysis

**Fig. 3:**
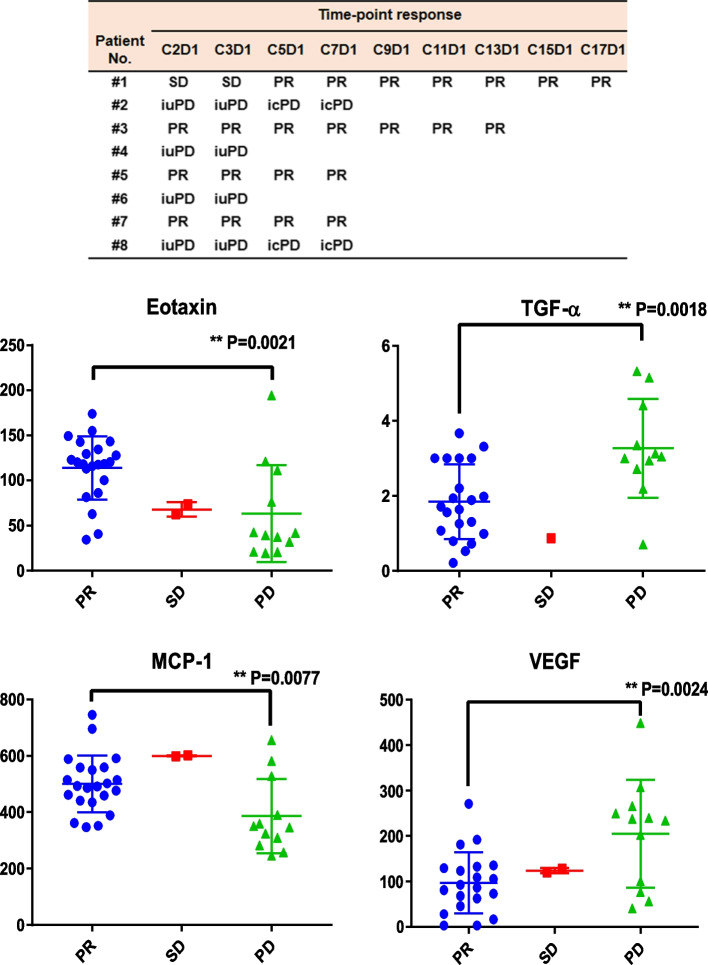
Potential biomarkers associated with efficacy. The responses at different time points when the cytokines were measured were showed in the upper panel. Levels of serum cytokines at different treatment cycles from 8 patients were analyzed against the corresponding response status. Among these cytokines, Eotaxin and MCP-1 levels showed a positive relationship with PR status, while TGF-alpha and VEGF levels correlated with PD (iuPD/icPD) status. Unpaired T- test was performed to analyze the difference of serum cytokines between PR and PD groups. *P* < 0.05 *, *P* < 0.01 **, *P* < 0.001 ***

We found that the levels of Eotaxin (CCL11) and MCP-1 (CCL2) were significantly higher when patients were at PR status compared to those at PD (iuPD/icPD) status. On the contrary, the levels of TNF-α and VEGF were significantly lower when patients were at PR status compared to those at PD status (as shown in Fig. 3). Other cytokines did not show increasing or decreasing patterns with response status.

## Discussion

In this study, Pucotenlimab achieved an ORR of 20.17% (24/119, 95% CI, 13.370%-28.506%) in ≥ second line setting, which was in line with the other anti-PD-1 products approved for advanced melanoma in a similar setting, among them were Pembrolizumab with an ORR of 16.7% (95% CI, 10.0%-25.3%) and Toripalimab with an ORR of 17.3% (95% CI, 11.2%–25.0%) [15, 16]. Unlike melanoma patients in the white population, which the cutaneous subtype dominates, the non-white patient population is mainly composed of acral or mucosal subtypes. Unfortunately, mucosal melanoma was usually refractory to treatment with a poor prognosis [17]. Compared with Toripalimab for mucosal melanoma with an ORR of 0% [15, 16], some patients with mucosal melanoma could benefit from Pucotelimab, as the ORR for this subgroup in this study was 8.7%.

The patients in the current study achieved a median OS of 16.59 (95% CI, 13.963–26.973) months, which was significantly longer than those of the conventional therapies of approximately 8 months [6]. According to the follow-up study after Pucotenlimab failure, merely 2 out of 55 patients achieved SD, and none achieved CR or PR from the follow-up treatments. Therefore, the longer mOS was contributed by Pucotenlimab to a great extent instead of the follow-up treatments.

In our study, the cutaneous subtype achieved the highest ORR among different known primary subtypes, followed by the acral, and then the mucosal subtype. The same patterns were observed in mPFS and mOS. Thus, ORR and PFS might be appropriate surrogate endpoints for OS, the gold-standard endpoint for efficacy in a similar setting.

ADA post Pucotenlimab treatment was detected in 4 out of 119 (3.36%). No ADA-related AE or aggravation was observed in these ADA-positive patients. Two of them achieved SD as the best response to Pucotenlimab (data was not showed). Therefore, it seemed that the influence of ADAs in the current study was minimal.

According to James C. Lee, et al., anti-P-1/PD-L1 therapy demonstrated low effectiveness for various cancers with liver metastasis including melanoma. The underlying mechanisms were CD8 + T cell depletion in liver and distal immunosuppression induced by regulatory T-cell activation [18]. Pucotenlimab also showed similar pattern. The ORR for the melanoma patients with liver metastasis was 6.67% (95% CI, 0.169%-31.948%), while for those without was 22.12% (95% CI, 14.566%-31.313%).

It was acknowledged that BRAF mutation did not affect the benefit melanoma patients got from anti-PD-1 treatment [19]. Our study also indicated that quite a proportion of patients (5/13, 38.46%) with BRAF mutation achieved CR/PR after Pucotenlimab treatment, which was comparable to that previously reported.

In our study, the rates of TRAEs of any Grade and of Grade ≥ 3 were 77.3% and 15.1%, respectively, which were similar to those reported in Pembrolizumab of 84.5% and 8.7%, and Toripalimab of 90.6% and 19.6% in the same setting. Besides, no new types of TRAEs appeared in our study compared with Pembrolizumab and Toripalimab during long-term safety follow-up [15, 16]. Therefore, the safety profile of HX008 was consistent with other anti-PD-1 antibodies.

In the current study, serum Eotaxin level during the treatment was positively correlated with PR status in patients treated with Pucotenlimab, which supported the efficacy-predicting role of Eotaxin and its effector cells eosinophil for anti-PD-1 treatment in various types of cancer including melanoma [20–24].

A variety of cancers (lung, breast, liver cancer) highly expressed CCL2, and usually it was associated with poor prognosis. Besides, Megan M. Tu, et al. showed that the efficacy of anti-PD-1 therapy was boosted by inhibiting CCL2 receptor, CCR2 [25]. Generally, CCL2 promotes tumor-growth through process, like inducing angiogenesis, recruiting MDSCs and metastasis-promoting monocytes, while it also has anti-tumor capability by driving tumor cell apoptosis [25, 26]. Interestingly, Tianqian Zhang, et al. revealed that CCL2 secreted by melanoma cells in 3-dimensional organoids as a Chemoattractant induced migration of cytotoxic T lymphocytes by CCR4 towards melanoma cells [27]. The prognostic role of CCL2 in melanoma has not been revealed yet. In the current study, it seemed that higher serum CCL2 level indicated a good outcome of Pucotenlimab treatment, which contradicted with the results in other cancers mentioned above. Thus, the evidence provided by Tianqian Zhang, et al. may support our opinion that CCL2 functioned as a good prognostic marker for PD-1 blockade in melanoma.

Previous studies showed that TGF-α was upregulated in many types of cancers including melanoma. Its higher serum level was associated with poorer prognosis. The underlying mechanism was its pro-differentiation capability for melanoma cancer cells [28]. Our study further supported this conclusion, as higher serum levels of TGF-α were detected when the patients were experiencing progressed disease.

Serum VEGF level has been suggested as a poor prognostic marker for Ipilimumab for the treatment of advanced melanoma, but not for PD-1 inhibitors or in combination with Ipilimumab [29]. Our study seemed to support VEGF serum levels as a poor prognostic factor for anti-PD-1 in the treatment of advanced melanoma.

Due to the limited sample size in the current study, the conclusions drawn above may be inaccurate, which needs further investigation in future phase III study that has been approved by the Chinese National Medical Products Administration (NMPA) with a large sample size involved.

## Conclusions

Pucotenlimab as a ≥ 2^nd^ line therapy showed promising efficacy and tolerable toxicity for patients with locally advanced or metastatic melanoma. Thus, it would broaden the treatment options for these patients. Meanwhile, the efficacy-predicting role of Eotaxin (CCL11), MCP-1 (CCL2), TNF-α, and VEGF for anti-PD-1 therapy was preliminarily justified. They might be developed as biomarkers helping decide whether to maintain or discontinue anti-PD-1 treatment.

## Data Availability

The datasets used and/or analyzed during the current study are available from the corresponding author on reasonable request.

## Abbreviations

PD-1: Programmed death-1
PD-L: PD-1 ligand
ORR: Overall response rate
DCR: Disease control rate
DOR: Duration of response
PFS: Progression-free survival
OS: Overall survival
IRC: Independent review committee
RECIST: Response Evaluation Criteria in Solid Tumors
TRAEs: Treatment-related adverse events
CTLA-4: Cytotoxic T-lymphocyte antigen 4
ECOG: Eastern Cooperative Oncology Group
AST: Aspartic transaminase
ALT: Alanine transaminase
CTCAE: National Cancer Institute Common Terminology Criteria for Adverse Events
ADA: Anti-drug-antibody
CR: Complete response
PR: Partial response
SD: Stable disease
PD: Progressed disease
FAS: The full analysis set
NMPA: The Chinese National Medical Products Administration
